# Using Dual-Site Transcranial Magnetic Stimulation to Probe Connectivity between the Dorsolateral Prefrontal Cortex and Ipsilateral Primary Motor Cortex in Humans

**DOI:** 10.3390/brainsci9080177

**Published:** 2019-07-26

**Authors:** Matt J.N. Brown, Elana R. Goldenkoff, Robert Chen, Carolyn Gunraj, Michael Vesia

**Affiliations:** 1 Department of Kinesiology and Health Science, California State University Sacramento, Sacramento, California, CA 95819-6073, USA; 2 Brain and Behavior Laboratory, School of Kinesiology University of Michigan, Ann Arbor, Michigan, MI 48109-2214, USA; 3 Division of Brain, Imaging and Behaviour—Systems Neuroscience, Krembil Brain Institute, Toronto, ON M5T 2S8, Canada; 4 Division of Neurology, Department of Medicine, University of Toronto, Toronto, ON M5G 2C4, Canada

**Keywords:** dorsolateral prefrontal cortex, primary motor cortex, transcranial magnetic stimulation, connectivity, motor control

## Abstract

Dual-site transcranial magnetic stimulation to the primary motor cortex (M1) and dorsolateral prefrontal cortex (DLPFC) can be used to probe functional connectivity between these regions. The purpose of this study was to characterize the effect of DLPFC stimulation on ipsilateral M1 excitability while participants were at rest and contracting the left- and right-hand first dorsal interosseous muscle. Twelve participants were tested in two separate sessions at varying inter-stimulus intervals (ISI: 4, 5, 6, 7, 8, 9, 10, 11, 12, 15, and 20 ms) at two different conditioning stimulus intensities (80% and 120% of resting motor threshold). No significant effect on ipsilateral M1 excitability was found when applying a conditioning stimulus over DLPFC at any specific inter-stimulus interval or intensity in either the left or right hemisphere. Our findings suggest neither causal inhibitory nor faciliatory influences of DLPFC on ipsilateral M1 activity while participants were at rest or when performing an isometric contraction in the target hand muscle.

## 1. Introduction

Motor and cognitive function depends on neural activity in a number of spatially distributed but interconnected brain regions. The dorsolateral prefrontal cortex (DLPFC), which includes Brodmann’s areas (BA) 46 and 9 [1,2], closely works with primary motor cortex (M1) to form fundamental circuitry involved in motor tasks of varying complexity [3]. Through its diverse anatomical projections to motor areas [4,5,6,7,8], DLPFC is a key node that mediates many higher-level aspects of motor control such as executive function [9], response selection [10], response initiation [11] and response inhibition [12]. A deeper knowledge of functional interactions between brain circuits and complex behavior is important to provide an essential foundation for understanding healthy brain function across the lifespan and neurological disorders [13]. Age-related declines in motor and cognitive performance often correspond with pathological changes in interregional connectivity within the motor system [14,15,16,17,18]. Likewise, brain networks associated with both cognitive and motor function are often impaired in many neurological disorders, including Parkinson’s disease, Alzheimer’s disease, and stroke [19,20]. It is therefore important to define DLPFC and motor interactions to understand the neurophysiological mechanisms required for complex goal-directed behavior and treatment of cognitive-motor impairments over the lifespan.

Neuroimaging indicates that cortical regions in the motor system such as premotor cortex (PMC), posterior parietal cortex (PPC), prefrontal cortex (PFC), and M1 are functionally linked and at least partially dependent on each other for motor control and complex behavior [21]. Dual-site transcranial magnetic stimulation (_ds_TMS) is a method to probe cortico-motor pathways both at rest and during different functional contexts in the motor system [22,23,24,25]. Specifically, this approach can evaluate the impact of a TMS conditioning stimulus (CS), applied over intra- or inter-hemispheric locations, on motor-evoked potentials (MEPs) elicited by the TMS test stimulus (TS) over M1.

Previous studies using _ds_TMS have investigated functional interactions between M1 and other motor related cortical areas, including dorsal premotor cortex (PMd) [26,27,28,29,30,31], ventral premotor cortex (PMv) [26,27,28,29,30], supplementary motor area (SMA) [27,32], posterior parietal cortex (PPC) [33,34,35,36,37,38,39,40] and somatosensory areas [41,42]. Facilitation (increased MEP amplitudes) and inhibition (reduced MEP amplitudes) are specific to the intensity, timing, and the functional context of the cortico-cortical pathway during stimulation [43] (for review see [25,44]). Of note, both intra- and inter-hemispheric input from frontal cortical areas to M1 change motor excitability for goal-directed behavior that requires a greater degree of cognitive control and executive function. Specifically, recent evidence using _ds_TMS indicates that stimulation of left DLPFC (BA 46) has a timing- and muscle- specific effect on ipsilateral motor cortical excitability in a choice reaction task [45]. Similarly, there is evidence for frontal control over contralateral motor cortex during action reprogramming, movement preparation and movement inhibition. In particular, the presupplementary motor area (pre-SMA), which is part of the medial frontal cortex and is known to have significant anatomical connections with DLPFC [2,46,47,48], has been shown to have a short-latency influence on M1 during action selection under conflict [49]. Likewise, inhibitory motor control depends on causal inhibitory influences of right prefrontal cortex (i.e., pre-SMA and interior frontal gyrus, IFG) on left motor cortical activity [50]. In addition, changes in DLPFC-M1 interactions are associated with decline in bimanual movements in older adults, suggesting greater reliance from nonprimary motor areas as task complexity increases [14].

However, one possible caveat that can confound the interpretation of these results relates to task-evoked activity superposed on top of resting-state activity [13]. Therefore, examining the timing of functional connectivity between DLPFC and M1 while participants are at rest can circumvent this confound and provide insight into their connections. Although inter-hemispheric interactions between frontal motor related cortical areas and M1 have been studied [51], it still remains to be shown whether DLPFC influences ipsilateral M1 excitability while participants are at rest or during isometric contraction of the target muscle. This fundamental knowledge has practical significance because DLPFC is currently being investigated as a possible target for treatment of cognitive-motor decline across the lifespan in both healthy and neurological disease states [52,53].

Here we investigate the effect of different interstimulus intervals (ISIs) and CS intensities applied over DLPFC on corticospinal excitability of ipsilateral M1 while participants were at rest for left (Experiments 1) and right (Experiments 3) hemispheres using _ds_TMS technique. We also examined whether an active contraction (compared to rest) is dissociable between DLPFC and ipsilateral M1 for left (Experiments 2) and right (Experiments 4) hemispheres. We hypothesized that CS intensities applied to DLPFC at rest would produce inhibition, whereas increasing excitability with an isometric muscle contraction would cause facilitation, similar to previous studies in other frontal cortical nodes in the motor network [29].

## 2. Materials and Methods

### 2.1. Participants

Twelve subjects (6 females, mean age 29.8 ± 6.8 SD years, range 21–44 years) participated in all four experiments after providing written informed consent. All participants were right-handed based on the Waterloo Handedness Questionnaire [54]. Data for the right hemisphere from one participant were removed from analyses because of an overly high level of muscle activity. None of the participants had any known contraindications to TMS [55]. All experimental procedures were approved by the University Health Network (Toronto) Research Ethics Board.

### 2.2. Electromyographic Recordings

Surface electromyograms (EMG) were recorded from the left and right first dorsal interosseous (FDI) muscles with 9 mm Ag-AgCl electrodes in a belly-tendon montage. Raw signals were amplified (×1000), band pass filtered (20 Hz–2.5 kHz; Intronix Technologies Corporation, Model 2024F, Bolton, Ontario, Canada), digitized at 5 kHz using a Micro 1401 data acquisition interface controlled by Signal Software version 7 (Cambridge Electronic Design Ltd, Cambridge, UK), and stored on a computer for off-line analysis.

### 2.3. Transcranial Magnetic Stimulation (TMS)

We used a paired-pulse _ds_TMS approach to test the functional interactions between DLPFC and ipsilateral motor cortex in each hemisphere. A CS was applied over DLPFC several milliseconds before applying a TS to the hand area of the primary motor cortex (M1_HAND_) that produces a MEP in the contralateral hand measured by EMG. As a result, the influence of CS from the first brain area (DLPFC) on the anatomically connected second area (M1_HAND_) can be tested by measuring the inhibitory and excitatory effects of MEP amplitude in the contralateral hand to measure functional interactions between a specific cortical area (such as DLPFC) and M1_HAND_ [22,23,24,44,56]. Four separate experiments were collected. Experiments 1 and 2 tested interactions between DLPFC and M1_HAND_ in the left hemisphere, while Experiments 3 and 4 tested DLPFC-M1_HAND_ connectivity in the right hemisphere on separate days. Experiments 1 and 3 were performed while participants kept both hands at rest. Experiments 2 and 4 were collected while participants held a ~20% maximum voluntary isometric contraction (MVC) in the right and left FDI muscle, respectively. To achieve this, we instructed participants to squeeze an object with their thumb and index finger to move a visual cursor between two horizontal lines, indicating the individual’s 20% MVC. Visual feedback of the cursors was displayed on an oscilloscope based on the level of force applied by the participant. Participants easily maintained the same relative spatial position of the cursor on the oscilloscope with very little reliance on the actual tracking cursor throughout the experiment. Importantly, participants reported that the continuous isometric contraction required a minimal amount of cognitive control to perform.

**Figure 1:**
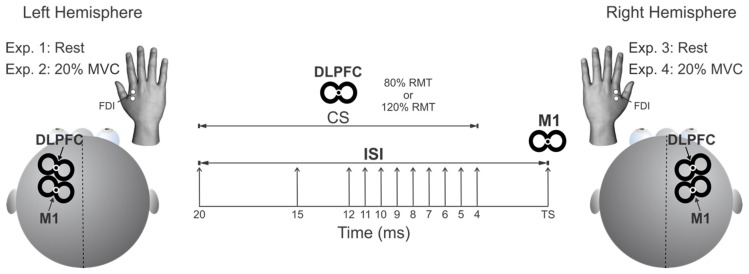
Experimental protocol. The conditioning stimulus (CS) coil was applied to the dorsolateral prefrontal cortex (DLPFC) and the test stimulus (TS) coil was applied to primary motor cortex (M1) in the left (Experiments 1 and 2) and right hemispheres (Experiments 3 and 4). DLPFC-ipsilateral M1 interactions were probed while participants were at rest or maintaining a 20% maximum voluntary contraction (MVC) of the left and right first dorsal interosseous (FDI) muscles. The CS intensity was 80% or 120% of resting motor threshold (RMT). The interstimulus interval (ISI) between pulses were 4, 5, 6, 7, 8, 9, 10, 11, 12, 15 or 20 ms.

Figure 1 illustrates the conditioning-test approach used for each of the four experiments. We applied the TS over M1_HAND_ with a small figure-of-eight branding iron coil (inner diameter: 50 mm) connected to a Magstim 200^2^ stimulator (Magstim Company, Whitland, UK). The TS coil over M1 was positioned at ~45° to the midsagittal plane to induce a posterior-to-anterior current within the cortex. We defined the optimum location of stimulation at which we could elicit MEPs of at least 50 µV peak-to-peak amplitude from the contralateral FDI muscles in the relaxed hand with the lowest stimulator intensity. We then adjusted the intensity of TS to elicit a peak-to-peak MEP amplitude of ~1 mV in FDI muscle for all experiments. We delivered an anterior-to-posterior current directed CS over DLPFC with a second figure-of-eight branding iron coil (inner diameter: 40 mm). For CS, we tested intensities of 80% and 120% of resting motor threshold (RMT) in all experiments. RMT in the left and right FDI muscles were defined as the lowest TMS intensity required to produce a MEP of >50 µV peak-to-peak amplitudes in 5 out of 10 consecutive trials with both hands at rest [57]. For all experiments, CS over DLPFC preceded TS over M1_HAND_ by ISI of 4, 5, 6, 7, 8, 9, 10, 11, 12, 15, and 20 ms. TMS [single (TS alone) and paired-pulse (CS-TS), ISI, and CS intensity] was randomized for each experiment. Stimuli were applied every 5 seconds. For each participant, twelve responses were collected for each ISI and CS intensity (a total of 288 trials) and averaged (16 blocks of 18 trials each). Measurements were repeated for each of the four experiments.

### 2.4. Localization of TMS Cortical Targets

**Figure 2:**
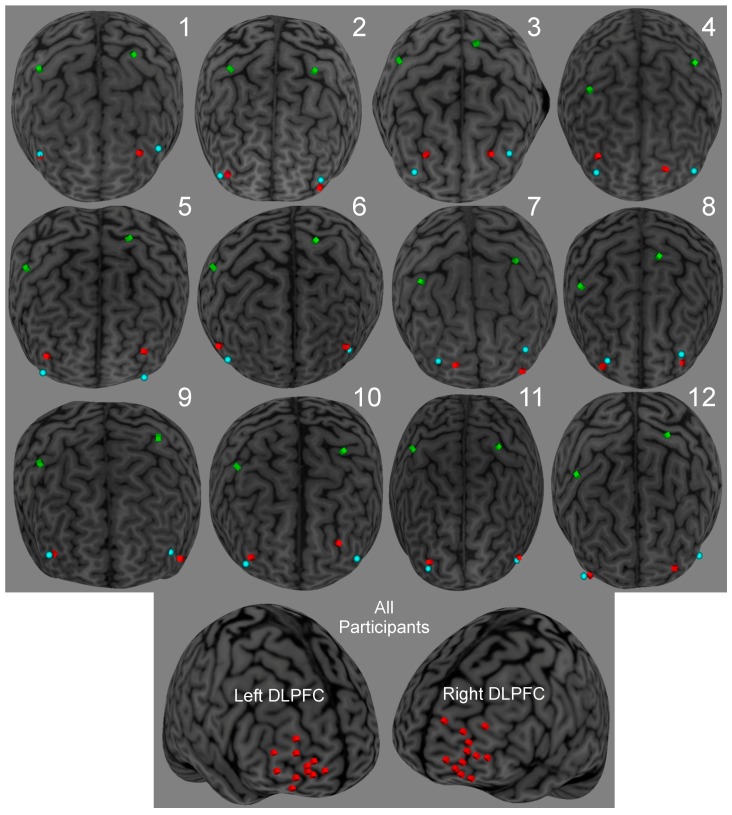
Dorsolateral prefrontal cortex (DLPFC) anatomical, DLPFC stimulation and M1 stimulation locations for Experiments 1–4 for the 12 participants based on individualized MRIs using neuronavigation software. We adjusted the targeted DLPFC stimulation sites to accommodate for both transcranial magnetic (TMS) coils on the scalp. Green cubes denote M1 stimulation locations, blue spheres denote DLPFC anatomical locations, and red cubes denote DLPFC stimulation locations.

We used methods previously described elsewhere [40]. Briefly, for left and right M1 we placed the TMS coil over the location where stimulation elicited the largest MEP from the contralateral FDI muscles. We then co-registered the scalp location with individual anatomical magnetic resonance imaging (MRI) scans (Brainsight, Rogue Research, Montreal, Quebec, Canada). Both left M1 (Talairach coordinates (TC); group mean ± SD, *n* = 12, *x* = −21.90 ± 7.31, *y* = −18.79 ± 10.57, *z* = 55.43 ± 5.66) and right M1 (*x* = 36.38 ± 5.47, *y* = −10.64 ± 10.56, *z* = 46.14 ± 6.11) sites overlapped with the ‘hand knob’ [58]. To target the DLPFC site, we selected individually determined anatomical landmarks for each participant along the middle frontal gyrus that corresponded to BA 46. This site was selected based on previous functional magnetic resonance imaging (fMRI) studies in humans showing activation for higher aspects of movement control such as response selection and movement attention [10,59,60]. Figure 2 shows the three-dimensional renderings of the structural MRI with marked cortical sites for all participants. Table 1 provides the anatomical and stimulation locations of the left and right DLPFC for each participant as well as the group mean and standard deviation.

### 2.5. Data Acquisiton and Analysis

To determine whether DLPFC stimulation modulates MEPs at rest or during an isometric contraction of the target muscle, separate one-way repeated measures analysis of variance (ANOVA_RM_) were performed for the left (Experiments 1 and 2) and right (Experiments 3 and 4) hemispheres on the MEP amplitudes with CS-TS interval (12 levels: 4, 5, 6, 7, 8, 9, 10, 11, 12, 15, 20 or TS alone) as the within-subject factor for both CS intensities (80% and 120% RMT) and contexts conditions (rest and 20% MVC). Next, we measured and expressed peak-to-peak amplitude of each MEP as a ratio of baseline control MEP (TS alone) for each participant. Normalized MEP amplitudes were then used in separate 2x11 ANOVA_RM_ for the left (Experiments 1 and 2) and right (Experiments 3 and 4) hemispheres using within-subject factors CS intensity (2 levels: 80% RMT or 120% RMT) and ISI (11 levels: 4, 5, 6, 7, 8, 9, 10, 11, 12, 15 or 20 ms) at rest and during the 20% MVC isometric contraction. The threshold for statistical significance was set at *p* ≤ 0.05.

## 3. Results

All participants tolerated the experimental procedures. The mean percentage of maximum stimulator output (MSO) for RMT with the CS coil and intensity required to elicit a MEP amplitude of ∼1 mV in the FDI muscle with the TS coil for each experiment is reported in Table 2. In all four experiments, no significant effect on ipsilateral M1 excitability (i.e., reduced or increased MEP amplitudes) was found when applying CS over DLPFC at any specific ISI or intensity.

For all four experiments we first performed a separate one-way ANOVA_RM._ In Experiment 1, we found a non-significant main effect of ISI on MEP amplitude for right FDI muscle for 80% RMT (F_(11,132)_ = 0.45, *p* = 0.93, η_p_^2^ = 0.04) and 120% RMT (F_(11,132)_ = 0.45, *p* = 0.93, η_p_^2^ = 0.02). In Experiment 2, we found a non-significant main effect of ISI on MEP amplitude for right FDI muscle for 80% RMT (F_(11,132)_ = 0.23, *p* ≥ 0.99, η_p_^2^ = 0.02) and 120% RMT (F_(11,132)_ = 0.30, *p* = 0.97, η_p_^2^ = 0.02). In Experiment 3, we found a non-significant main effect of ISI on the MEP amplitude for left FDI muscle for 80% RMT (F_(11,120)_ = 0.21, *p* ≥ 0.99, η_p_^2^ = 0.02) and 120% RMT (F_(11,120)_ = 0.22, *p* ≥ 0.99, η_p_^2^ = 0.02). In Experiment 4, we found a non-significant main effect of ISI on the MEP amplitude for left FDI muscle for 80% RMT (F_(11,120)_ = 0.32, *p* = 0.98, η_p_^2^ = 0.03) and 120% RMT (F_(11,120)_ = 0.33, *p* = 0.98, η_p_^2^ = 0.03).

**Figure 3:**
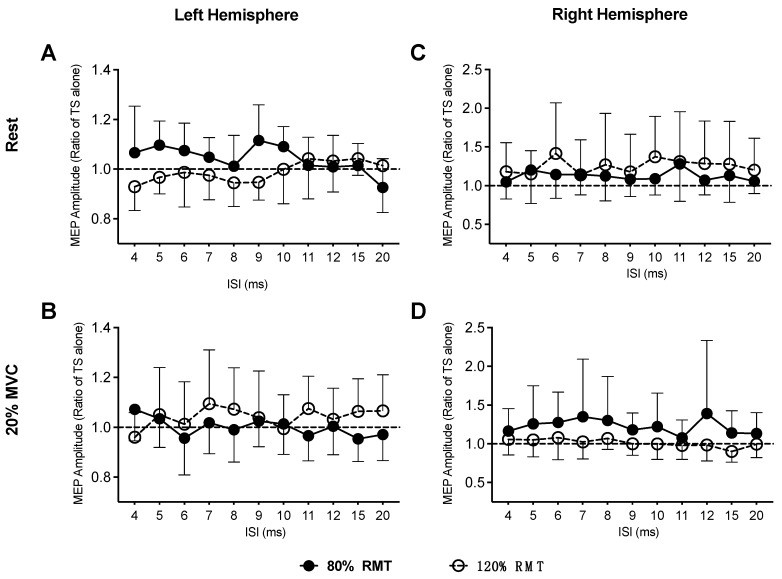
Normalized motor evoked potential (MEP) amplitudes for at rest condition (**A**) or during a 20% maximum voluntary contraction (MVC) of the target muscle (**B**) in the left hemisphere and at rest condition (**C**) or during a 20% MVC of the target muscle (**D**) in the right hemisphere. Ratios below one represent inhibition, whereas ratios above one denote facilitation. Results revealed that MEP amplitudes were not significantly inhibited nor facilitated at both CS intensities and all ISIs. Error bars represent 95% confidence intervals.

To directly compare the patterns of DLPFC-M1 functional connectivity observed under the two different CS intensities, we normalized MEP amplitudes to TS alone in each participant for each CS intensity, and then plotted the mean group ratio for MEP amplitude (ordinate) as a function of ISI (abscissa) for FDI muscle. Figure 3 plots the normalized MEP amplitudes for DLPFC-M1 interactions for all four experiments. A two-way ANOVA_RM_ revealed a non-significant main effect of CS intensity (F_(1,11)_ = 1.38, *p* = 0.27, η_p_^2^ = 0.11), ISI (F_(10,110)_ = 0.59, *p* = 0.82, η_p_^2^ = 0.05), and their interaction (F_(10,110)_ = 1.43, *p* = 0.12, η_p_^2^ = 0.61) on the MEP amplitude ratio for right FDI muscle at rest (Figure 3A, left hemisphere). Similarly, we found a non-significant main effect of CS intensity (F_(1,11)_ = 0.60, *p* = 0.46, η_p_^2^ = 0.11), ISI (F_(10,110)_ = 0.38, *p* = 0.95, η_p_^2^ = 0.03), and their interaction (F_(10,110)_ = 1.23, *p* = 0.28, η_p_^2^ = 0.10) on the MEP amplitude ratio for right FDI muscle while participants maintained a muscle contraction (Figure 3B). For the right hemisphere, we also found a non-significant main effect of CS intensity (F_(1,10)_ = 1.18, *p* = 0.30, η_p_^2^ = 0.17), ISI (F_(10,100)_ = 1.48, *p* = 0.16, η_p_^2^ = 0.71), and their interaction (F_(10,100)_ = 1.39, *p* = 0.19, η_p_^2^ = 0.67), on the MEP amplitude ratio for left FDI muscle at rest (Figure 3C). Same results were found with a 20% MVC for the right hemisphere, as was revealed by a non-significant main effect of CS intensity (F_(1,10)_ = 1.22, *p* = 0.29, η_p_^2^ = 0.17), ISI (F_(10,100)_ = 1.07, *p* = 0.39, η_p_^2^ = 0.10), and their interaction (F_(10,100)_ = 0.47, *p* = 0.91, η_p_^2^ = 0.23) on the MEP amplitude ratio for left FDI muscle during the 20% MVC (Figure 3D).

## 4. Discussion

We used a conditioning-test _ds_TMS approach to examine the temporal and functional interactions of frontal cortico-cortical circuits, focusing on inputs to M1 from ipsilateral DLPFC while participants were at rest or contracting the target muscle. The current findings provide evidence for no causal inhibitory nor facilitatory influences of DLPFC (BA 46) on ipsilateral M1 activity while participants were at rest, irrespective of hemisphere. They also imply that the transmission of causal influence of DLPFC on ipsilateral M1 activity does not change during sustained isometric contractions in the target muscle. Recent _ds_TMS studies strongly suggest that the influence of frontal cortical areas on motor cortical excitability are associated with cognitive control of movements. For example, changes in functional connectivity between left DLPFC (BA 46) and ipsilateral M1 in the non-target and, to a lesser extent, the target hand muscle have been found during movement preparation of cued and free selection tasks [45]. In particular, increased left DLPFC-M1 connectivity at an ISI of 12 ms was found at different times during movement preparation for visually-cued and free selection tasks [45]. In another study, increased right pre-SMA-left M1 connectivity at 6 and 12 ms was found during movement preparation during trials that involved switching responses [49]. In addition, increased right pre-SMA-left M1 and right IFG-left M1 connectivity coincided with NoGo trials at an ISI of 6 ms, suggesting prefrontal control over motor cortex during the preparatory phase of movement inhibition [50]. Importantly, the increased intra-hemispheric DLPFC-M1 and inter-hemispheric pre-SMA-M1 and IFG-M1 connectivity were all found during the movement preparation phase for tasks requiring executive control (i.e., a task switching response), action selection, action initiation or action inhibition. Our experiments did not examine connectivity during movement preparation, but rather during an isometric contraction, which could explain why we did not find increased DLPFC-M1 interactions. Together with these previous findings, our results imply two things. First, it shows that the DLPFC may have minimal impact on ipsilateral M1 excitability at rest and during a simple motoric task. Second, it shows that frontal cortical areas influence M1 excitability only when these neural pathways are engaged in more complex movements that require greater cognitive control. Given the prominent role of DLPFC in proactive inhibitory control [12], future studies should explore intra-hemispheric functional interactions between the DLPFC and M1 as task complexity increases during movement inhibition [50] and bimanual movement control [14].

Our study has several methodological limitations. First, we only examined two CS intensities. Future experiments should examine a wider range of CS intensities, similar to previous work [36,51], to determine whether specific CS intensities could selectively activate neurons in the said neural pathway. Second, it is possible that the optimal DLPFC site influencing motor excitability varies between participants. Because we targeted the DLPFC site according to individually determined anatomical landmarks for each participant, this could have introduced a large interindividual variability of the optimal cortical area for inducing an effect with the CS on motor excitability. Therefore, it would be interesting to examine the relative contribution of different locations close to our targeted frontal cortex using fMRI task-based localizers [61]. Third, the position and orientation of the TMS coil over DLPFC was fixed in our experiments. Future work should examine different TMS coil orientations to induce directionally-specific electric fields in the brain to selectively target specific frontal-motor circuits. Fourth, future studies should employ larger sample sizes. Finally, there is limited evidence for direct monosynaptic connections between the DLPFC and ipsilateral M1 [4,5,6,7,8]. Anatomically, the DLPFC is connected to premotor areas within BA 6 [2,47], but may preferentially target the pre-SMA and rostral portions of the dorsal premotor cortex (pre-PMd) [46,47,48]. DLPFC also is known to have reciprocal connections with the basal ganglia [12,62,63] and connections to various motor nuclei within the thalamus [7,64,65,66]. These polysynaptic connections provide multiple pathways for the DLPFC to causally influence M1 excitability. However, our _ds_TMS technique is unable to specifically target any of these potential pathways. Therefore, conditioning DLPFC stimulation could cause complex activity in these multiple polysynaptic pathways, which may be of particular importance to explain our lack of DLPFC-M1 resting-state connectivity findings with _ds_TMS. Interestingly, contralateral DLPFC stimulation (defined as 5 cm anterior to M1) has been shown to modulate M1 excitability at longer ISIs (i.e., 30, 40, 50 and 60 ms) across a wider range of CS intensities [52]. Therefore, it is possible that the timing for measuring functional connections between DLPFC and ipsilateral M1 at rest could be at longer ISIs than those measured in the current experiment. Although we hypothesized that intra-hemispheric interactions between DLPFC and ipsilateral M1 should occur at shorter ISIs than the previously reported inter-hemispheric interactions [52], it is possible that this hypothesis is incorrect. Furthermore, the intra-hemispheric effects between DLPFC and M1 at rest might occur via inter-hemispheric cortico-cortical connections that, in turn, influence M1 at longer ISIs. Future research should examine the role of inter-hemispheric cortico-cortical connections on intra-hemispheric DLPFC-M1 connectivity using TMS [67].

## 5. Conclusions

In summary, our data show for the first time that stimulation of DLPFC does not influence ipsilateral motor cortex activity both while participants were at rest and contracting the target muscle. Although caution should be used when interpreting findings with TMS [68], it would be fruitful if future work uses the conditioning-test _ds_TMS approach described here to probe how DLPFC-M1 interactions dynamically change during higher-level aspects of motor control across the lifespan and how these interactions are affected by abnormalities of motor and cognitive function in pathological conditions such as stroke, Parkinson’s, and Alzheimer’s disease.

## Figures and Tables

**Table 1:** Left and Right dorsolateral prefrontal cortex (DLPFC) anatomical and actual transcranial magnetic stimulation (TMS) stereotaxic neuronavigated locations based on Talairach coordinates (*x*, *y*, *z*) in mm based on Talairach Atlas.

Participant	Left DLPFC						Right DLPFC					
	Anatomical Location			Stimulation Location			Anatomical Location			Stimulation Location		
	*x*	*y*	*z*	*x*	*y*	*z*	*x*	*y*	*z*	*x*	*y*	*z*
1	−32.61	46.64	20.29	−31.89	49.39	15.74	35.64	40.82	19.71	30.14	44.27	25.78
2	−39.74	30.10	22.41	−27.39	39.06	29.32	38.59	33.28	22.85	38.46	33.86	22.04
3	−28.89	26.58	21.29	−17.91	31.44	28.36	26.52	35.2	18.03	19.28	30.38	28.54
4	−31.33	45.31	18.36	−12.87	54.64	36.09	29.68	45.89	23.71	28.51	39.2	32.86
5	−34.37	45.32	11.64	−32.48	39.82	32.12	33.4	51.22	15.74	31.81	46.05	27.2
6	−29.4	38.34	17.5	−27.67	41.07	24.79	33.65	46.6	15.22	37.82	34.51	16
7	−26.02	45.86	18.75	−24.3	52.63	5.25	24.86	50.07	14.13	15.04	56.61	16.78
8	−32.13	43.11	16.13	−38.18	38.16	7.57	33.02	44.88	13.76	29.75	44.15	14.5
9	−25.33	51.12	21.38	−25.15	50.72	13.62	22.69	50.8	19.22	25.85	53.87	17.16
10	−36.71	47.95	12.28	−25.27	52.59	31.31	32.48	55.01	16.6	29.09	55.67	23.03
11	−34.97	44.93	12.01	−36.03	44.00	13.72	25.87	55.16	18.6	24.73	55.68	25.36
12	−39.76	32.76	1.26	−23.92	50.13	14.39	33.76	47.5	5.82	29.81	46.04	4.44
Group Mean ± SD	−32.61 ± 4.77	41.50 ± 7.76	16.11 ± 6.01	−26.92 ± 7.18	45.30 ± 7.36	21.02 ± 10.46	30.85 ± 4.87	46.37 ± 7.00	16.95 ± 4.71	28.36 ± 6.68	45.02 ± 9.18	21.14 ± 7.73

**Table 2:** Mean percentage of maximum stimulator output (MSO) required to elicit resting motor threshold (RMT) with the 40 mm conditioning stimulus (CS) coil and 1mV motor evoked potentials (MEPs) with the 50 mm test stimulus (TS) coil during rest and isometric muscle contraction. Data are shown for both the left and right primary motor cortex (M1) for each participant and group mean and standard deviation.

Participant	Left M1			Right M1		
	RMT	Rest 1 mV	Active 1 mV	RMT	Rest 1 mV	Active 1 mV
1	47	58	47	49	49	40
2	45	56	45	44	50	46
3	58	75	62	57	58	45
4	53	60	52	54	58	47
5	50	68	45	37	39	34
6	51	56	45	51	54	41
7	47	65	56	51	63	52
8	52	70	54	53	72	56
9	45	49	42	39	39	35
10	59	70	62	62	73	59
11	41	45	39	52	52	42
12	50	51	45	50	55	48
**Group Mean ± SD**	49.8 ± 5.3	60.3 ± 9.4	49.5 ± 7.6	49.9 ± 7.1	55.2 ± 10.8	45.4 ± 7.7
